# Pneumovirus-Induced Lung Disease in Mice Is Independent of Neutrophil-Driven Inflammation

**DOI:** 10.1371/journal.pone.0168779

**Published:** 2016-12-22

**Authors:** Bart Cortjens, René Lutter, Louis Boon, Reinout A. Bem, Job B. M. van Woensel

**Affiliations:** 1 Pediatric Intensive Care Unit, Emma Children’s Hospital AMC, Amsterdam, The Netherlands; 2 Experimental Immunology and Respiratory Medicine, Academic Medical Center, Amsterdam, The Netherlands; 3 Epirus Biopharmaceuticals Netherlands, Utrecht, The Netherlands; University of North Carolina at Chapel Hill, UNITED STATES

## Abstract

The human pneumovirus respiratory syncytial virus (RSV) is the most common pathogen causing lower respiratory tract disease in young children worldwide. A hallmark of severe human RSV infection is the strong neutrophil recruitment to the airways and lungs. Massive neutrophil activation has been proven detrimental in numerous diseases, yet in RSV the contribution of neutrophils to disease severity, and thereby, the relevance of targeting them, is largely unknown. To determine the relevance of potential neutrophil targeting therapies, we implemented antibody-mediated neutrophil depletion in a mouse pneumonia virus of mice (PVM) model. PVM is a host specific murine pneumovirus closely related to human RSV, which reproduces many of the features of RSV infection, such as high viral replication and neutrophil recruitment. Clinical disease and markers of lung inflammation and injury were studied in PVM-infected mice treated with either depleting or isotype control antibodies. To confirm our results we performed all experiments in two mice strains: C57Bl6 and BALBc mice. Neutrophil depletion in blood and lungs was efficient throughout the disease. Remarkably, in both mouse strains we found no difference in clinical disease severity between neutrophil-depleted and control arms. In line with this observation, we found no differences between groups in histopathological lung injury and lung viral loads. In conclusion, our study shows that in mice neutrophil recruitment to the lungs does not affect disease outcome or viral clearance during severe PVM infection. As such, this model does not support the notion that neutrophils play a key role in mouse pneumovirus disease.

## Introduction

The human pneumovirus respiratory syncytial virus (RSV) is the most common cause of bronchiolitis in young children worldwide [1–3]. Global mortality is estimated at almost 200,000 deaths per year in children under the age of 5 years, but occurs mainly in resource-limited countries [1]. In the US alone, 172,000 children are admitted annually to the hospital due to severe RSV disease and approximately 10% of these children need mechanical ventilation to survive [3]. Many of these mechanically-ventilated children fulfil the clinical criteria for acute respiratory distress syndrome (ARDS) at some point during their admission [4]. Currently, there is no licensed vaccine or effective therapeutic treatment for human RSV disease. It is imperative to gain more insight into the key pathogenic mechanisms of human RSV infection in order to develop new therapeutics.

One of the key features seen during human RSV infection is influx of neutrophils into the airways and alveolar compartment of the lungs. Up to 76% of the cells present in the airways and lungs are neutrophils [5]. Additionally, post-mortem examination of lung tissue sections from fatal RSV cases predominantly shows neutrophilic infiltration with airway obstruction caused by neutrophil-rich mucus plugs [6]. Strong lung neutrophil recruitment is also observed in animal pneumovirus disease, such as bovine RSV infection in calves and pneumonia virus of mice (PVM) in rodents [7]. Both human and animal pneumovirus infections elicit prominent CXC chemokine responses, including CXCL-8 [8, 9] and KC [10], which likely contribute to the neutrophilic inflammation. However, so far it is unknown if these high numbers of neutrophils in the airways and lungs are solely protective or may also be detrimental during pneumovirus infections.

On the one hand, neutrophils possess a broad arsenal of defensive strategies, including reactive oxygen species (ROS)-production, phagocytosis, release of toxic granule contents and the formation of neutrophil extracellular traps (NETs) [11]. Several of these neutrophil anti-microbial mechanisms have been proven to be effective against viruses, including human RSV [12, 13]. In addition, neutrophils have also been shown to take up RSV virions, suggesting a contributive role to viral clearance [14]. On the other hand, the mechanisms by which neutrophils kill microorganisms can also induce collateral damage to the host. For example, extensive neutrophil accumulation is considered a key mechanism in the development of the diffuse lung endothelial and epithelial injury observed in ARDS [15]. A similar injurious role of neutrophils in RSV disease has been proposed before [12, 16]. Indeed, several markers of neutrophilic inflammation correlate with (peak) RSV disease severity [12]. In addition, our group recently showed aggravated NET formation in mucus plugs in the airways of bovine RSV-infected calves, contributing to airway obstruction [13]. As such, preventing neutrophil-induced damage and/or airway obstruction could improve disease outcome in RSV disease.

Experimental neutrophil depletion in mice can be used to increase our insight in the precise role of neutrophils in host defense or pathogenesis of (respiratory) viral diseases. However, the results of many studies vary substantially, depending on type and strain of virus, animal model (e.g. C57Bl6 or BALBc mice) and depletion regime used [17–20]. For example, influenza infection in neutrophil depleted mice with the low virulent strain BJx109 does not alter disease outcome, while in the same mice lethal PR8 influenza virus infection leads to enhanced lung injury and uncontrolled viral replication [21], indicating differences in neutrophil-virus interactions between strains. Importantly, Stokes et al. previously showed that neutrophil depletion during human RSV infection in BALBc mice results in reduced histopathological damage, but does not affect viral clearance [22]. However, in contrast to cognate host pneumovirus models, there is a relatively mild lung neutrophilic response upon infection by human RSV in mice [7]. In addition, the clinical signs of illness and viral replication in this heterologous model are limited. Therefore, in the present study we aimed to determine the role of neutrophils in a relevant host-pneumovirus interaction by using antibody-mediated neutrophil depletion in PVM infected mice. We hypothesized that neutrophils are detrimental during severe PVM disease and as such, that neutrophil depletion could result in improved clinical and histopathological outcomes.

## Materials and Methods

### Virus

PVM virus strain J3666 was used in all experiments and was originally obtained from Dr. A.J. Easton (University of Warwick, Coventry, UK) from a virus stock originating at the Rockefeller University. Virulence was maintained by continuous passage in mice [23].

### Animal protocol

All animal protocols were approved by the Institutional Animal Care and Use Committee of the University of Amsterdam, the Netherlands, the results are reported according to the ARRIVE guidelines. The mice were maintained under specific pathogen‐free conditions according to local guidelines. Eight week‐old female C57Bl6 (Charles River, Leiden, The Netherlands) and BALBc mice (Envigo, Horst, The Netherlands) were used. After arrival the animals were randomly allocated to isocages, (5–6 animals/cage) and allowed to acclimatize for 7 days prior to start of the experiment with food and water *ad libitum*. Further details on animal housing and baseline animal characteristics are provided in the S1 supplemental methods and S1 table. On day 0, all animals were infected with 2.3 × 10^4^ copies of PVM diluted in 80 μL RPMI-1640 (Gibco, Grand Island, NY, USA) by intra-nasal inoculation under 2% isoflurane inhalation anesthesia. At the end of the experiment, euthanasia was performed by intraperitoneal injection of pentobarbital. The left lung was removed and flash‐frozen in liquid nitrogen for homogenization. The right lung was lavaged three times with NaCl 0.9%/EDTA 0.6 mmol/L (initial volume of 0.6 mL, followed by two times 0.5 mL) and the obtained broncho-alveolar lavage fluid was pooled. After the lavage, the lung was removed and fixed in 10% formalin for histological studies, while in a separate group of mice the lungs were not lavaged prior to fixation (as indicated at the appropriate figures).

### Neutrophil depletion

Neutrophil depletion was achieved by intraperitoneal injection of 500 μg specific anti-Ly6G monoclonal antibody (1A8 mAb, Bio X Cell, West-Lebanon, USA) [24] every 48 hours, starting in the morning, one day before PVM infection (day -1) until the end of the experiment (6 mice/group). The control group was treated with isotype control antibodies (Rat IgG2a, 6 mice/group). Blood neutrophil depletion was confirmed by tail-vein blood smear analysis obtained immediately before every next antibody injection. In separate ‘semi-survival’ experiments, in which the follow up of mice was extended to 12 days (C57Bl6 mice, 5 mice/group), the mice received either anti-Ly6G or isotype control antibodies every 48 hours during the first 7 days, after which daily injections were given to adequately suppress neutrophil numbers.

### Clinical response

Our primary outcome was total body weight and clinical score. These were obtained daily as described previously [25]. The endpoint for sacrifice used in this study was > 20% weight loss and/or a clinical score ≥ 4.

### Lung virus load

Viral loads were determined in lung tissue by qPCR, detecting the number of copies of the PVM *sh* gene (GenBank No. AY573815). RNA was isolated from frozen lungs using the RNeasy Mini Kit (Qiagen, Venlo, the Netherlands). Two μg of RNA was reverse transcribed to cDNA using random hexamers (high‐capacity cDNA reverse transcription kit; Applied Biosystems, Foster City, CA). Total number of *sh*-copies was detected in qPCR reactions containing 1 μL of cDNA, Sybrgreen PCR Master Mix (Applied Biosystems) and 250 nmol/L primers (5′‐GCCGTCATCAACACAGTGTGT‐3′ and 5′‐GCCTGATGTGGCAGTGCTT‐3′). The *gapdh* housekeeping gene was detected in cDNA samples using rodent *gapdh* primers (250 nmol/L, Ambion, Austin, TX). Standard curves with known concentrations of the full‐length *sh* gene and *gapdh* decatemplate (Applied Biosystems) were used for quantification. Results are expressed as *sh* copies per 10^9^ *gapdh* copies.

### Lung histology

Standard haematoxylin and eosin‐stained lung sections were prepared and evaluated under a standard light microscope. In order to test interstitial neutrophil depletion the lungs were stained with an anti-mouse Ly6C/G monoclonal antibody (Rb6-8C5, BD Pharmingen, San Diego, CA). Neutrophil extracellular trap formation in lung tissue was assessed using staining with anti-citrullinated histone H3 (Ab5103, Abcam, Cambridge, UK) as described before [13] in non-lavaged lung sections of C57Bl6 mice and BALBc mice.

### BAL measurements

Total cell counts in BAL was measured with the Coulter Counter (Beckman Coulter, Brea, CA, USA). A sample containing 3 × 10^4^ BAL cells was added to cytospins (Shandon cytospin 3, Thermo Scientific, Rockford, IL, USA) and were manually differentiated by counting 400 cells in a blinded fashion. The following cytokines were measured in BAL by magnetic Luminex assay (R&D, Minneapolis, MN, USA): IFN-y, KC, MIP-1α, IL10, IL17A, IL6. Lung permeability was determined by measuring the high-molecular-weight protein IgM in BAL, a large protein that is absent in BAL of non-infected mice, by ELISA (Bethyl Laboratories, Montgomery, TX, USA), as described before [25]. BAL neutrophil degranulation was measured by myeloperoxidase (MPO) ELISA (DY3667, R&D). ROS-production was measured by quantitative western blot for detection of 4-Hydroxynenal (4HNE, AB5605, Millipore, Darmstadt, Germany) in BAL normalized for protein content by Bradford assay. 4HNE is produced under influence of ROS [26].

### Statistical analysis

Statistical analysis was performed using Graphpad Prism 5. Results are presented as median with interquartile range (IQR) or mean ± SD. Results between groups were compared using the Mann-Whitney U-test or Log-rank test were appropriate. We used a 95% confidence interval to determine significance.

## Results

### 1A8 mAb efficiently depletes neutrophils in blood and lungs

**Fig 1:**
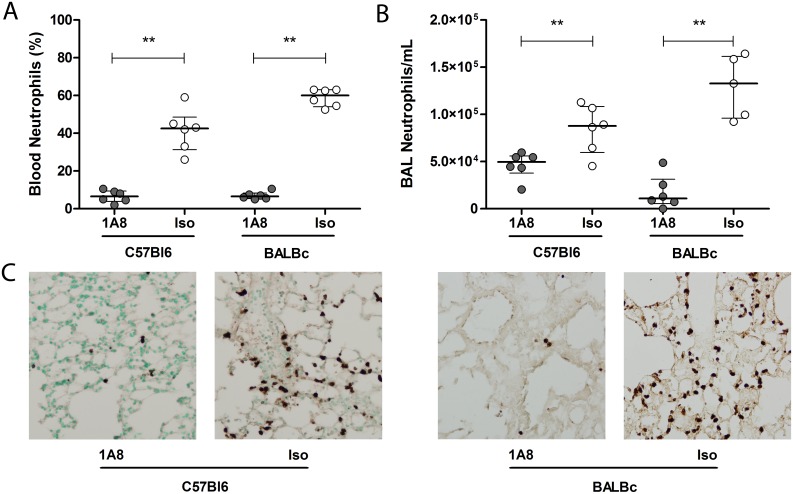
Neutrophil depletion during severe PVM disease in mice. Percentage of neutrophils present in blood **(A)** and total number of neutrophils per mL of BAL **(B)** in C57Bl6 and BALBc mice treated with either 1A8 mAb (grey circles, N = 6/group) or isotype control antibody (white circles, N = 6/group), measured on day 8 (C57Bl6, blood p = 0.002, BAL p = 0.009) and day 7 (BALBc, blood p = 0.002, BAL p = 0.008) after PVM inoculation. **(C)** Representative images of Ly6C/G-staining of lung tissue sections showing minimal interstitial neutrophil numbers in the 1A8 mAb treated animals on day 8 (C57Bl6) and day 7 (BALBc) after inoculation (magnification 200×). Data are shown as individual values and median with bars depicting IQR. ** p < 0.01

To investigate neutrophils during severe pneumovirus disease we first performed a viral dose titration experiment to determine the optimal viral inoculum dose. We determined a dose of 2.3 × 10^4^ copies of PVM as the minimal inoculum inducing severe respiratory disease, thereby avoiding the use of an excess viral dose, thus minimizing direct viral-induced cytopathology (S1 Fig). In this severe pneumovirus disease model, neutrophil depletion by the specific anti-Ly6G 1A8 mAb resulted in significant depletion in blood and BAL in both C57Bl6 and BALBc mice (Fig 1A and 1B). Neutrophil depletion in blood remained constant throughout the course of PVM disease (S2 Fig). Lung interstitial depletion of neutrophils in the mice treated with 1A8 mAb was confirmed by Ly6C/G staining of lung tissue sections in both mice strains (Fig 1C). Non-infected animals receiving either 1A8 mAb or isotype control antibodies did not show signs of disease or weight loss (S3 Fig).

### Neutrophil depletion does not alter PVM clinical disease

**Fig 2:**
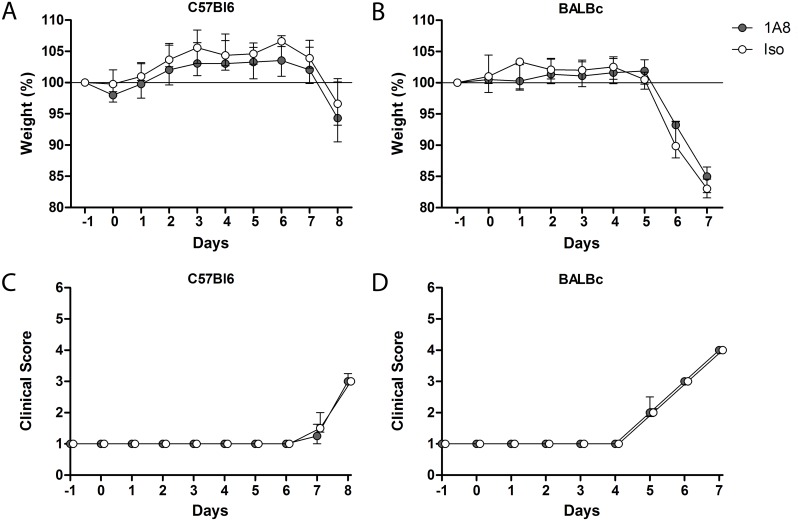
Clinical PVM disease severity. **(A-B)** Weight loss and clinical score of illness as measured by the modified Cook’s score **(C-D)** [25] in C57Bl6 and BALBc mice treated with either 1A8 mAb (grey circles, N = 6) or isotype control antibody (white circles, N = 6) during the course of severe PVM disease. No significant differences between groups. Data are shown as median with bars depicting IQR.

Next, we evaluated weight loss and clinical score on a daily basis to detect early and late clinical signs of PVM infection with and without neutrophil depletion. Both the 1A8 mAb and isotype control antibody treated C57Bl6 mice deteriorated equally, starting from day 6–7 after inoculation: there were no differences in the progression of clinical scores and weight loss (Fig 2A and 2C). Importantly, these findings were confirmed in BALBc mice, which are more susceptible to PVM and have more pronounced neutrophil recruitment [7, 27]. The BALBc mice indeed succumbed more early, starting at day 4–5 after inoculation, but similarly to the C57Bl6 mice, no differences in weight loss or clinical scores were seen between neutrophil depleted and isotype control treated BALBc mice (Fig 2B and 2D).

**Fig 3:**
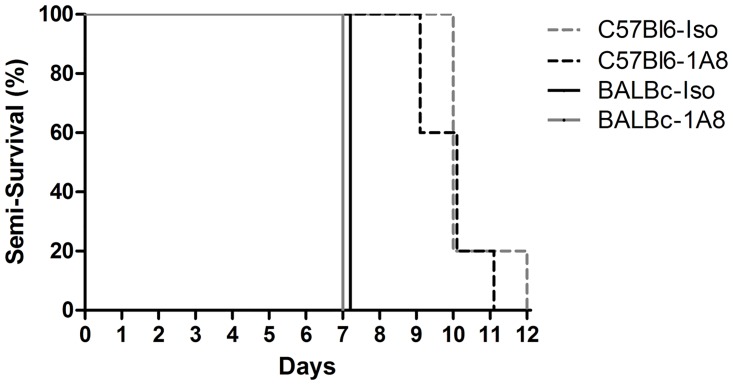
Semi-survival experiment. Kaplan-Meier curves showing the percentages of BALBc mice (solid lines) and C57Bl6 mice (dashed lines) treated with either 1A8 mAb (black, N = 5-6/group) or isotype control antibody (grey, N = 5-6/group) reaching the end point of a clinical score of ≥ 4 and/or > 20% weight loss after PVM inoculation (not significant).

In additional experiments we prolonged the duration of follow-up to test if neutrophil depletion would alter end-stage rather than early clinical symptoms. In line with our previous experiments, PVM induced severe disease in both strains, with end-stage disease in BALBc mice on day 7 and in C57Bl6 mice on day 9 to 12 after inoculation, independent of neutrophil depletion (Fig 3).

### Neutrophil depletion does not affect PVM replication or histopathological lung injury

**Fig 4:**
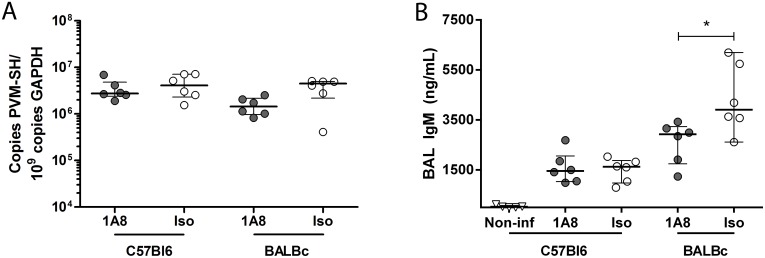
Viral load and lung permeability. **(A)** Viral loads in viral copies per 10^9^ GAPDH copies in C57Bl6 (day 8) and BALBc mice (day 7), no significant differences between 1A8 mAb treated (grey circles, N = 6/group) or isotype control treated animals (white circles, N = 6/group). **(B)** Lung permeability as measured by IgM (ng/mL) in BAL of C57Bl6 (non-infected and PVM infected, day 8) and PVM infected BALBc mice (day 7). Increased IgM levels after PVM infection, with a significant increase in isotype control treated (white circles, N = 6/group) BALBc mice, compared to 1A8 mAb treated (grey circles, N = 6/group) BALBc mice (* p = 0.03). Data are shown as individual values and median with bars depicting IQR.

**Fig 5:**
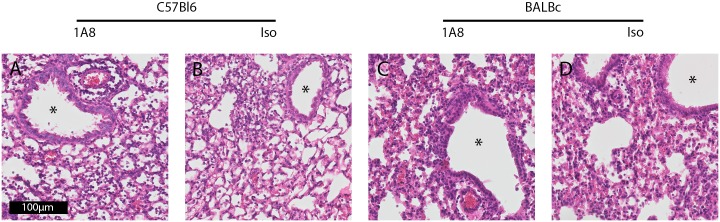
Lung histopathology. **(A,C)** Representative image of HE-staining of C57Bl6 mice (day 8), showing interstitial cellular infiltrates and proteinacious debris **(B,D)** HE-staining of BALBc mice (day 7), showing hemorrhaging and proteinacious debris (magnification 400×).

**Fig 6:**
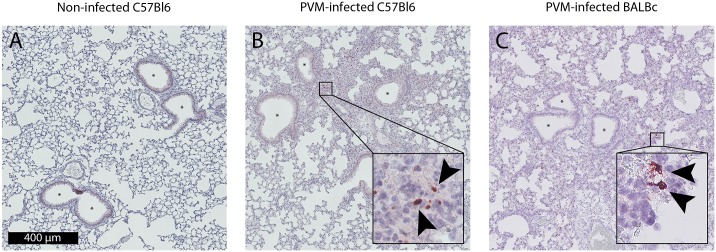
NET production. **(A)** Citrullinated histone H3 staining of a non-infected lung tissue section, no NETs are visible (magnification 200×). **(B,C)** Citrullinated histone H3 staining of PVM infected C57Bl6 (day 8) and BALBc (day 7) mice (non-lavaged lung sections, magnification 200×) shows scarce NET formation (insets, magnification 1200×) without airway occlusion (asterisk).

To determine if neutrophils influence viral clearance we measured the PVM load in lung tissue. We found no significant differences in viral load between neutrophil depleted and isotype control treated groups for both the C57Bl6 and BALBc strains (Fig 4A). As reported before, severe PVM disease was associated with elevated levels of IgM in BAL of C57Bl6 and BALBc animals, indicating enhanced lung permeability [25, 28]. The IgM levels were not different between the neutrophil depleted and the isotype control treated C57Bl6 mice, whereas levels were significantly higher in isotype control antibody treated BALBc mice as compared to 1A8 mAb treated mice (Fig 4B, p = 0.03). Histopathology showed severe lung injury by PVM infection with peri-bronchial and alveolar cellular infiltrates, deposition of intra-alveolar proteinaceous debris and mild alveolar septal thickening (C57Bl6 and BALBc mice), and capillary congestion with areas of hemorrhage (BALBc mice) (Figs 5 and 6). However, this was consistent in both neutrophil depleted and control mice. In line with previous reports [25, 29], we observed very limited mucus plugs obstructing the airways in both the C57Bl6 and BALBc mice during PVM infection. To assess one of the specific effector functions of neutrophils recently implicated in severe RSV disease [13], we also evaluated the formation of NETs during severe PVM infection. NETs were scarce in this model, with evidence for a limited number of NETs observed almost exclusively in the most severely injured alveolar areas (Fig 6A–6C). This is in sharp contrast with findings in influenza infection, where NETs were prominent and contributed to alveolar-capillary injury [19].

### Neutrophil depletion alters the lung cytokine profile

Neutrophil depletion did not significantly alter the recruitment of macrophages and lymphocytes towards the lungs during severe PVM disease (Table 1). In contrast, we did find a significant increase in the concentration of KC in BAL in 1A8 mAb-treated animals compared to control mice (C57Bl6: 153.1 ± 37.8 vs 73.7 ± 19.2 pg/mL, p = 0.002 and BALBc: 489.9 ± 152.0 vs 218.9 ± 91.1 pg/mL, p = 0.026, Table 1). Furthermore, local IL6 levels were significantly higher in 1A8 mAb-treated C57Bl6 animals, but not in BALBc mice. Levels of MIP-1α, IL10 and IFN-γ were not significantly different between the treatment groups (Table 1). BAL MPO content as a marker of neutrophil degranulation, was elevated after PVM infection (Table 1) and significantly lowered in neutrophil depleted BALBc mice compared to isotype control mice (Table 1, p = 0.03). ROS-production was absent in non-infected animals and reduced in neutrophil depleted animals (Table 1, p = 0.03).

**Table 1:** BAL cellularity, cytokines and neutrophil activation.

	Control C57Bl6 (day 8)	C57Bl6 (day 8)			BALBc (day 7)		
	Non-infected	1A8	Iso	p = *	1A8	Iso	p = *
**BAL Cellularity(10**^**e**^**5/mL)**							
Total cells	2.4 (1.85–3.20)	6.00 (4.11–7.23)	4.81 (2.99–5.10)	**0.13**	5.15 (2.58–7.47)	3.17 (2.99–4.50)	**0.90**
Neutrophils	ND	0.5 (0.38–0.56)	0.88 (0.60–1.09)	**0.009**	0.13 (0.04–0.37)	1.33 (0.96–1.61)	**0.008**
Macrophages	2.4 (1.85–3.20)	4.34 (3.23–5.56)	3.08 (2.00–3.43)	**0.24**	3.67 (1.92–5.69)	1.39 (1.30–2.59)	**0.06**
Lymphocytes	ND	1.01 (0.50–1.13)	0.60 (0.39–0.71)	**0.13**	0.96 (0.45–1.80)	0.24 (0.19–0.94)	**0.19**
**BAL cytokines(pg/mL)**							
KC	5.0 ± 4.1	153.1 ± 37.8	73.7 ± 19.2	**0.002**	489.9 ± 152.0	218.9 ± 91.1	**0.026**
MIP-1α	0.3 ± 0.3	24.8 ± 7.9	45.0 ± 19.6	**ns**	31.11 ± 19.3	31.1 ± 6.6	**ns**
IL10	0.9 ± 1.4	64.7 ± 57.3	69.5 ± 38.8	**ns**	212.4 ± 125.4	159.1 ± 78.6	**ns**
IL6	ND	703.4 ± 274.1	351.2 ± 63.7	**0.02**	1728 ± 1217	2178 ± 1542	**ns**
IL17A	ND	ND	ND		ND	ND	
IFN-γ	ND	1183 ± 941.9	1014 ± 475.7	**ns**	829.4 ± 653.1	775.2 ± 195.4	**ns**
BAL MPO(ng/mL)	ND	141 ± 36	123 ± 37	**ns**	135 ± 57	232 ± 71	**0.03**
BAL 4HNE(Intensity)	0.7 ± 0.5	4.9 ± 2.7	7.4 ± 3.1	**ns**	5.8 ± 1.9	9.0 ± 2.3	**0.03**

ND; not detectable, ns; not significant, data depicted as median/interquartile range or mean ± SD.

*; p values were calculated using Mann-Whitney U-test and represent the comparison between 1A8 treated and isotype control mice.

## Discussion

The main goal of this study was to determine the role of neutrophils during severe PVM disease in a relevant natural host-pathogen interaction. In this work, we show that although neutrophil depletion is associated with moderately altered lung cytokine and permeability responses, neutrophils do not play a major role in defining clinical illness and histopathological lung injury in mice infected with a lethal inoculum of PVM. In addition, neutrophil depletion does not affect viral clearance in this mouse model.

Although neutrophilic inflammation is originally associated with bacterial infections, current insights point towards a similar role for neutrophils during viral infections [30]. Importantly, several studies suggest a protective role of neutrophils during viral respiratory infections. For example, neutrophil depletion by 1A8 mAb during moderate and severe influenza virus infection in mice leads to increased mortality and lung injury with higher viral loads, indicative of a protective and anti-viral role for neutrophils [18, 21]. Follow up studies show that this protective effect was the result of early regulatory functions of neutrophils, as late depletion (> 5 days after infection) did not improve survival [17, 31]. Studies with BAL and blood neutrophils isolated from RSV-infected children have shown that neutrophils take up RSV particles, which suggests that neutrophils either contribute to the clearance of RSV or that they may support viral replication [14]. In addition, activated neutrophils release NETs upon exposure to RSV *in vitro*, which may under certain circumstances act as a defense mechanism against viral binding and infectivity of target cells [13]. However, so far *in vivo*, no studies have actually shown that neutrophils play an important role in controlling RSV infection. For example, in a study by Stokes *et al*. it was found that lung viral load remains unaltered upon neutrophil depletion in RSV infection in mice [22]. These results are in line with our findings, that neutrophils are not involved in the clearance of PVM infection in mice.

Alternatively, neutrophilic inflammation may have major adverse effects during respiratory viral disease. Enhanced or prolonged activation of neutrophil effector functions (e.g. ROS-production, NETs, degranulation) is considered a key event in the development of alveolar-capillary damage during ARDS [15, 32]. Again, in the influenza virus infection model, Brandes et al. have shown that mice have increased survival after *partial* neutrophil depletion [18]. They found that early pro-inflammatory feed-forward circuits lead to excess neutrophil-mediated inflammatory damage during fatal influenza infection. These results indicate that neutrophils can already exert its essential innate host defense functions in small numbers, but aggravated recruitment tips the balance towards development of lung injury. Although neutrophil depletion in human RSV-infected mice showed that neutrophils mediate mucin expression and thereby potentially contribute to airway obstruction [22], *in vivo* models studying the effects of neutrophilic inflammation on lung permeability and injury during PVM infection were lacking. Our results indeed showed decreased lung permeability in neutrophil depleted BALBc mice during PVM infection. However, in our model the extent of this damage did not translate into alterations in lung histopathology nor in noticeable differences in the clinical outcome.

Remarkably, despite moderate changes in the local cytokine profile and lung permeability by 1A8 mAb treatment, neutrophils were found to have a very limited role in either host defense or immunopathology in our PVM mouse model. To confirm our initial findings in C57Bl6 mice we choose to evaluate a more susceptible strain: BALBc mice. The outcomes were consistent in both mouse strains. A similar minor effect of neutrophil depletion has been reported in studies using intranasal herpes simplex virus infection and under certain conditions during mild influenza virus infection as mentioned above [20, 21]. Still our findings were highly unexpected, given the profound neutrophil recruitment and activation observed during pneumovirus infections, including PVM infection in mice (in particular in the highly susceptible BALBc mice) [19–22]. There may be several explanations for the lack of a significant effect on clinical response and viral clearance in this study. First, clinical disease in our severe pneumovirus model may, for a large part, be dependent on direct virus-induced cytopathology. This is in line with studies by Vincenzo et al. who showed that viral load drives clinical disease in humans with hRSV infection [33, 34]. Similarly, this effect has been shown in influenza infection in mice, where high viral inoculum led to comparable disease between CD200^-/-^ and WT mice opposed to significant differences between groups infected with lower viral inocula [35]. PVM is particularly virulent and airway and alveolar epithelial cells of mice support rapid replication of PVM [7]. Both CD4^+^ and CD8^+^ T cells independently contribute to immunopathology and viral clearance in mild PVM infection. Interestingly, T cell-deficient mice succumb equally fast after inoculation with a lethal dose of PVM compared to WT mice [36]. This underlines the viral dose-dependency of clinical illness in PVM infected mice. As such, our study adds to this insight by showing that neutrophils are not involved in defining the outcome in severe PVM infection.

Second, we observed relatively little mucus obstruction in the airways during PVM infection. This is in contrast to the profound airway mucus plugs seen in RSV disease in children [37]. Recently, we have shown extensive NET formation in these plugs [13], implicating neutrophils in the development of airway obstruction during severe RSV disease. Interestingly, hardly any NET formation was seen during severe PVM infection in mice, which can be regarded as an important shortcoming of this model for human RSV disease. Although important differences in neutrophil effector functions between humans and mice are known [38], the relative paucity of NET formation in the PVM model appears unrelated to this species issue, as other reports have shown extensive NET formation during respiratory viral infection in mice [19]. Thus, the absence of NETs and mucus plugs in PVM-infected mice could partially explain the lack of effect of neutrophil depletion in our study. In addition, PVM infection results in strong neutrophil responses in mice, however the absolute and relative amount of neutrophils at baseline and peak-disease remain lower compared to the human situation [5, 38]. As such, these results cannot be readily extrapolated to human RSV disease.

Finally, in this study we have utilized the Ly6G-specific 1A8 mAb to deplete neutrophils. In contrast to the widely used RB6-8C5, which depletes not only Ly6G^+^ (neutrophils) but also Ly6C^+^ cells (e.g. Ly6C^+^ monocytes), 1A8 mAb-mediated depletion is proven neutrophil specific [20, 21]. Though depletion is specific, it is not 100% complete: in line with previous reports [20, 21] there remains a baseline level of circulating blood neutrophils (± 6%) and BAL neutrophils (± 2%), despite increasing the frequency of 1A8 mAb administration. The remaining regulatory and/or destructive capabilities of this small pool of neutrophils, residing in a heightened pro-inflammatory environment with elevated local levels of KC and IL6, may have compensated for the major neutrophil deficiency. However, markers of degranulation and ROS-production in particular neutrophil depleted BALBc mice were low in our study, which disagrees with such a scenario, and even then it would be highly unlikely that this would not translate into any (partial) difference in outcome.

In conclusion, our study shows that neutrophils do not have a major role in disease outcome and viral clearance during PVM infection in mice. This finding is remarkable as PVM infection was shown to be associated with strong neutrophil recruitment and activation. As such, this rodent specific pneumovirus model does not support the notion that neutrophils play a key role during severe mouse pneumovirus disease. Because of important differences in neutrophil dynamics and function between mice and humans, as exemplified by the relative absence of NET formation during PVM infection, these results cannot be readily extrapolated to human RSV disease. Future studies in humans and other animal models must extend these findings and further address the role of neutrophils in human RSV disease.

## Supporting Information

S1 Fig *In vivo* PVM dose titration. C57Bl6 mice (N = 3/group) were inoculated on day 0 with the viral dose as indicated in the graph. All animals were monitored up to 10 days. Animals were culled after reaching the end point of a clinical score of > 4 and/or > 20% weight loss after PVM inoculation. Data are shown as median with bars depicting IQR.
(TIF)

S2 Fig Blood neutrophils percentages during 1A8 mAb treatment. Blood neutrophil percentages in C57Bl6 mice (A) and BALBc mice (B) either treated with 1A8 mAb (solid dots, N = 6/group) or isotype control antibodies (open dots, N = 6/group). Data are shown as median with bars depicting IQR.
(TIF)

S3 Fig Weight loss during 1A8 mAb or isotype control treatment. Weight loss in non-infected C57Bl6 mice treated with either 1A8 mAb (filled dots, N = 5) or isotype control antibody (open dots, N = 4). No signs of disease or weight loss were registered. Data are shown as median with bars depicting IQR.
(TIF)

S1 Supplemental Method Animal housing and handling. (DOC)

S1 Table Baseline animal characteristics. Iso; isotype control antibodies, 1A8; 1A8 monoclonal antibody, Weight in grams ± SD.
(DOC)
