# Supplementary figures and images for: Pneumovirus-Induced Lung Disease in Mice Is Independent of Neutrophil-Driven Inflammation
Source: PLoS One. 2016 Dec 22;11(12):e0168779. doi: 10.1371/journal.pone.0168779 (PMC5179008; doi:10.1371/journal.pone.0168779)

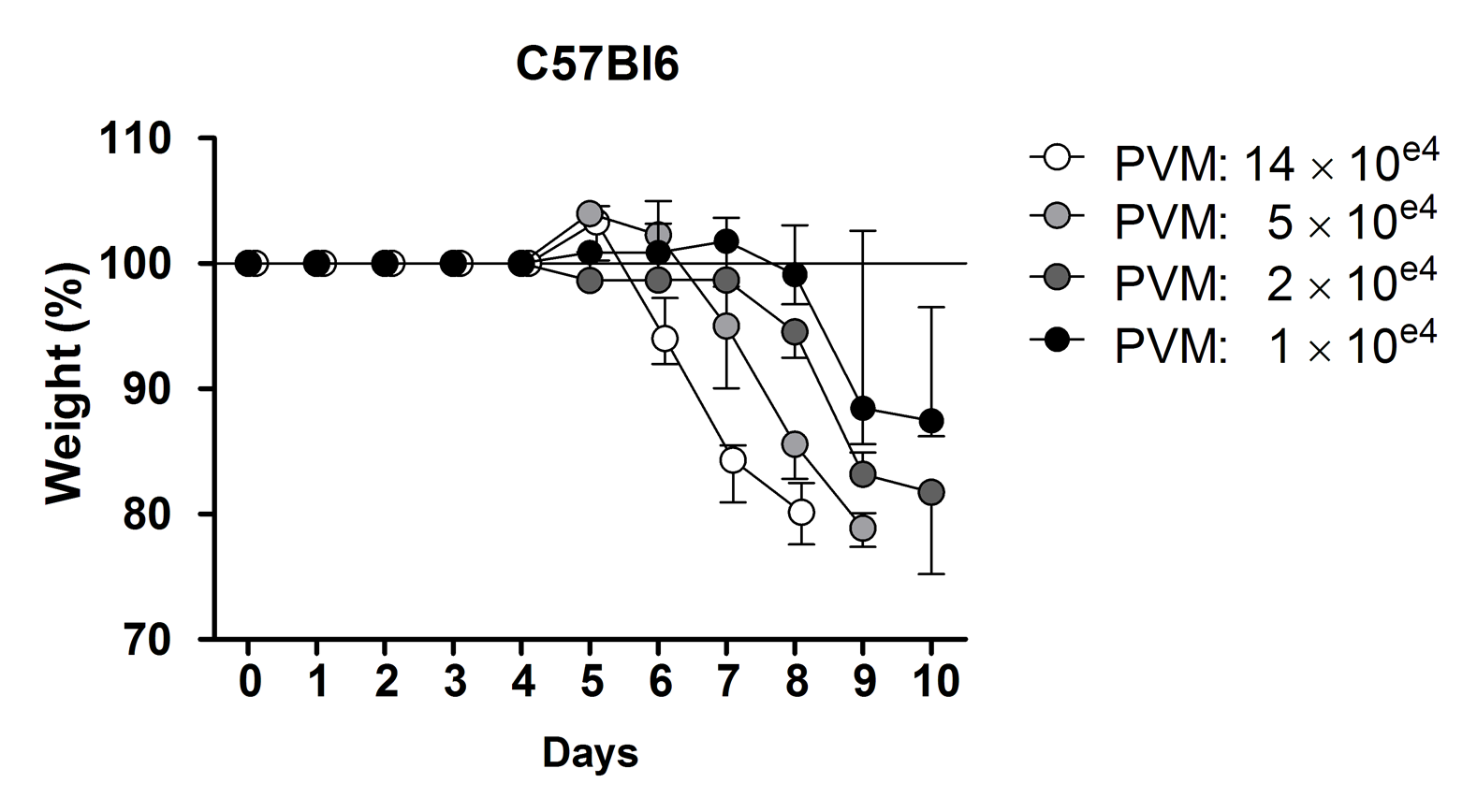

Supplement: S1 Fig — C57Bl6 mice (N = 3/group) were inoculated on day 0 with the viral dose as indicated in the graph. All animals were monitored up to 10 days. Animals were culled after reaching the end point of a clinical score of > 4 and/or > 20% weight loss after PVM inoculation. Data are shown as median with bars depicting IQR. (TIF) [file pone.0168779.s001.tif]

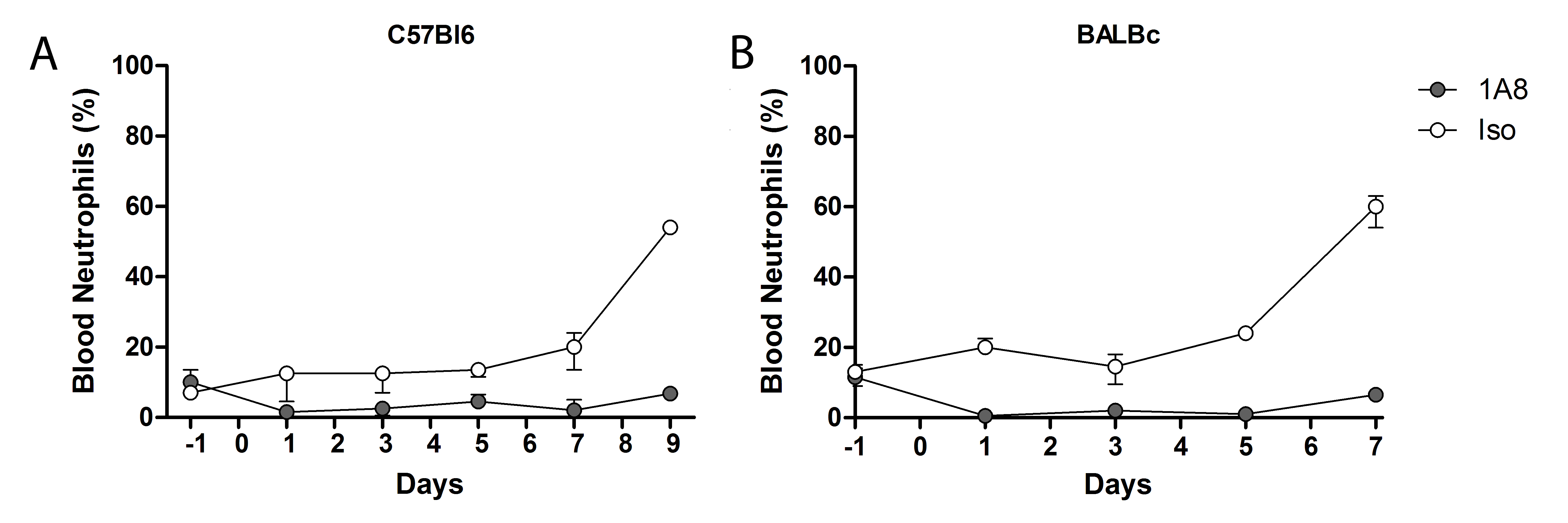

Supplement: S2 Fig — Blood neutrophil percentages in C57Bl6 mice (A) and BALBc mice (B) either treated with 1A8 mAb (solid dots, N = 6/group) or isotype control antibodies (open dots, N = 6/group). Data are shown as median with bars depicting IQR. (TIF) [file pone.0168779.s002.tif]

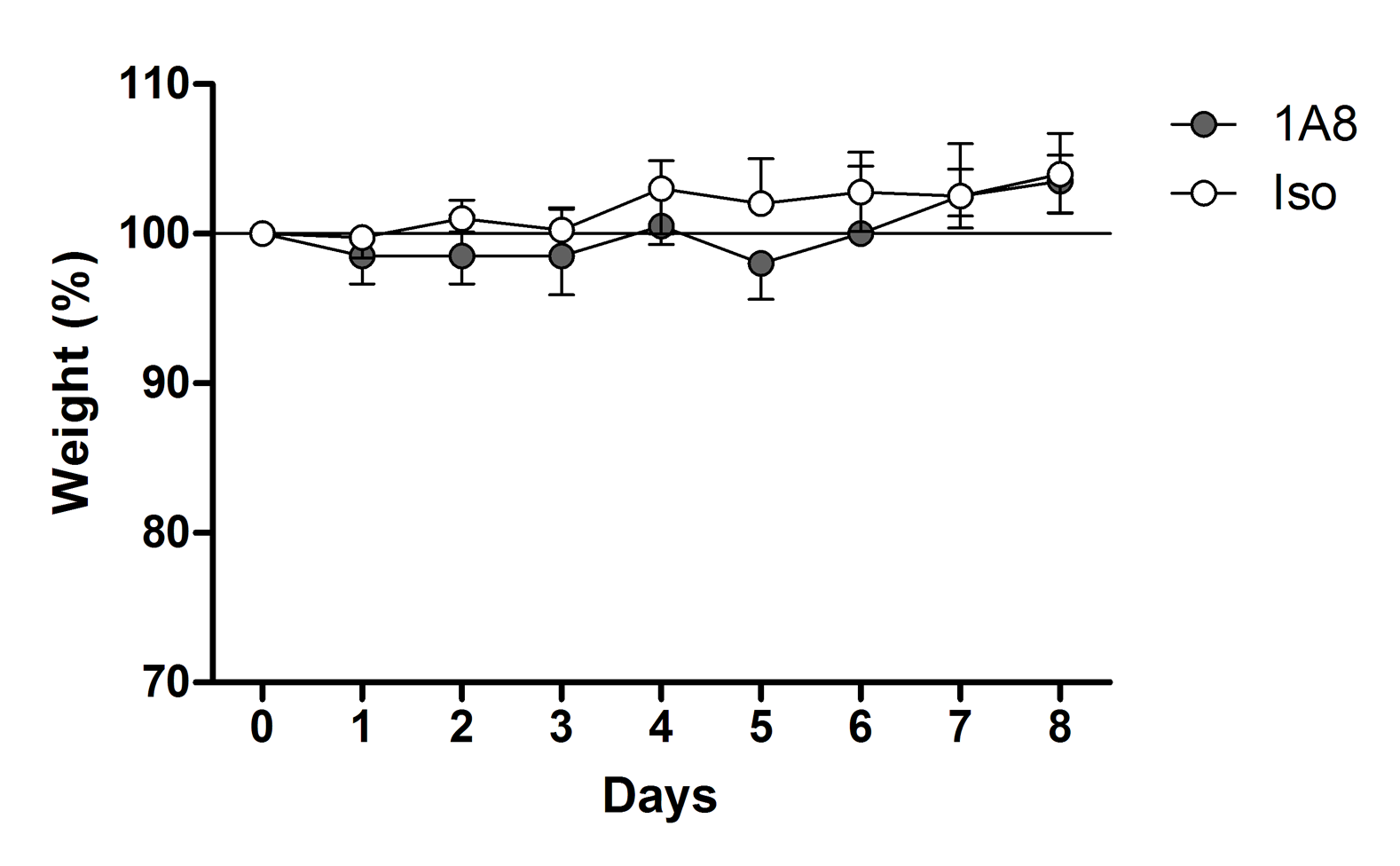

Supplement: S3 Fig — Weight loss in non-infected C57Bl6 mice treated with either 1A8 mAb (filled dots, N = 5) or isotype control antibody (open dots, N = 4). No signs of disease or weight loss were registered. Data are shown as median with bars depicting IQR. (TIF) [file pone.0168779.s003.tif]
