# Supplementary material for: Pneumovirus-Induced Lung Disease in Mice Is Independent of Neutrophil-Driven Inflammation
Source: PLoS One. 2016 Dec 22;11(12):e0168779. doi: 10.1371/journal.pone.0168779 (PMC5179008; doi:10.1371/journal.pone.0168779)
Supplement: S1 Supplemental Method — (DOC) [file pone.0168779.s004.doc]

**S1 Supplemental Method**

**Animal housing and handling**

All animals were housed in isocages (Tecnilab, Someren, The Netherlands, pressure -20kPa). Animals were housed with 5-6 mice per cage and maintained at ± 20°C, 40-70% relative humidity with 75 air-changes per hour. The animal room had 11 hours of artificial light per day, starting at 7 am. Bedding consisted of wood chips, changed once a week. Cages were enriched with cardboard houses and tissues. Nutrition (CRM, Tecnilab-BMI) *ad libitum*, water (acidified, pH 2.6-2.9) *ad libitum*, both changed every week. The mice were not handled during acclimatization. During the experiments all mice were handled once daily for weight monitoring and every second day for intraperitoneal injection.
