# Supplementary material for: Pneumovirus-Induced Lung Disease in Mice Is Independent of Neutrophil-Driven Inflammation
Source: PLoS One. 2016 Dec 22;11(12):e0168779. doi: 10.1371/journal.pone.0168779 (PMC5179008; doi:10.1371/journal.pone.0168779)
Supplement: S1 Table — Iso; isotype control antibodies, 1A8; 1A8 monoclonal antibody, Weight in grams ± SD. (DOC) [file pone.0168779.s005.doc]

**S1 Table: Baseline animal characteristics**

| **Baseline data** | **Strain** | **Sex** | **Age** | **Treat-ment** | **Weight (g)** | **Infection** | **N / N analyzed (on primary outcomes)** | **Reason not analyzed** |
| --- | --- | --- | --- | --- | --- | --- | --- | --- |
| **Exp. 1 Depletion efficacy in healthy mice** | C57BL/6NCrl (SPF) | Female | 8 wks | Iso | 19.8 ± 0.2 | No | 5/4 | Died prematurely due to arterial puncture |
|  |  |  |  | 1A8 | 20.3 ± 0.5 | No | 5/5 |  |
| **Exp. 2 Viral titration** | C57BL/6NCrl (SPF) | Female | 8 wks | None | 21.5 ± 1.0 | Yes | 14/14 |  |
| **Exp. 3 Day 8** | C57BL/6NCrl (SPF) | Female | 8 wks | Iso | 20.6 ± 0.5 | Yes | 6/6 |  |
|  |  |  |  | 1A8 | 19.9 ± 0.3 | Yes | 6/6 |  |
| **Exp. 4 Semi-survival** | C57BL/6NCrl (SPF) | Female | 8 wks | Iso | 20.5 ± 1.1 | Yes | 5/5 |  |
|  |  |  |  | 1A8 | 20.1 ± 0.7 | Yes | 5/5 |  |
| **Exp. 5 Day 7 and Semi-survival** | BALB/cOlaHsd (SPF) | Female | 8 wks | Iso | 19.5 ± 0.4 | Yes | 6/6 |  |
|  |  |  |  | 1A8 | 18.7 ± 0.5 | Yes | 6/6 |  |
|  |  |  |  | None | 20.0 ± 1.0 | Yes | 4/4 |  |

Iso; isotype control antibodies, 1A8; 1A8 monoclonal antibody, Weight in grams ± SD
